# Fat Sensation: Fatty Acid Taste and Olfaction Sensitivity and the Link with Disinhibited Eating Behaviour

**DOI:** 10.3390/nu9080879

**Published:** 2017-08-15

**Authors:** Sophie Kindleysides, Kathryn L. Beck, Daniel C. I. Walsh, Lisa Henderson, Shakeela N. Jayasinghe, Matt Golding, Bernhard H. Breier

**Affiliations:** 1 School of Food and Nutrition, Massey Institute of Food Science and Technology, College of Health, Massey University, Auckland 0745, New Zealand; s.j.kindleysides@massey.ac.nz (S.K.); k.l.beck@massey.ac.nz (K.L.B.); henderson.lisama@gmail.com (L.H.); S.N.Jayasinghe@massey.ac.nz (S.N.J.); M.Golding@massey.ac.nz (M.G.); 2 Nano Consulting Limited, Auckland 0632, New Zealand; daniel@nanoconsulting.co.nz

**Keywords:** fatty acid taste, olfaction, sensory, mouthfeel, test-retest, eating behaviour, dietary intake, BMI, taste

## Abstract

Perception of fat taste, aroma, and texture are proposed to influence food preferences, thus shaping dietary intake and eating behaviour and consequently long-term health. In this study, we investigated associations between fatty acid taste, olfaction, mouthfeel of fat, dietary intake, eating behaviour, and body mass index (BMI). Fifty women attended three sessions to assess oleic acid taste and olfaction thresholds, the olfactory threshold for *n*-butanol and subjective mouthfeel ratings of custard samples. Dietary intake and eating behaviour were evaluated using a Food Frequency and Three-Factor Eating Questionnaire, respectively. Binomial regression analysis was used to model fat taste and olfaction data. Taste and olfactory detection for oleic acid were positively correlated (*r* = 0.325; *p* < 0.02). Oleic acid taste hypersensitive women had significantly increased *n*-butanol olfactory sensitivity (*p* < 0.03). The eating behaviour disinhibition and BMI were higher in women who were hyposensitive to oleic acid taste (*p* < 0.05). Dietary intake of nuts, nut spreads, and seeds were significantly correlated with high olfactory sensitivity to oleic acid (*p* < 0.01). These findings demonstrate a clear link between fatty acid taste sensitivity and olfaction and suggest that fat taste perception is associated with specific characteristics of eating behaviour and body composition.

## 1. Introduction

Taste is the sensation experienced when a chemical stimulus or tastant in the mouth is recognised by receptors of the taste buds. There are five established taste modalities including sweet, salty, sour, bitter, and umami (savoury). Sweet, umami, and bitter molecular sensors have been identified as G-protein coupled receptors (GPCRs), salt is recognised by an ion channel receptor (ENaC) and the sour taste receptor mechanism (yet to be identified) responds to the presence of acid [1,2,3].

Fat creates a range of textural qualities which are considered to be the well-known sensory properties of fat, such as creaminess, oiliness, and thickness [4,5]. Until recently, fat was considered only to be perceived through mouthfeel and olfaction, but there is now considerable evidence that fatty acids can be perceived by specific taste receptors of the tongue [6,7,8]. Several studies have demonstrated varying gustatory sensitivities to fatty acids at low concentrations [9,10,11,12]. Furthermore, detection of volatile fatty acids by odour [13,14] or mouthfeel have been implicated in enhancing enjoyable eating experiences [15]. 

Increasingly, taste is being investigated for its role in the signaling pathways which govern the body’s response to incoming food [16]. The physiological mechanisms of taste have multiple functions which include: signaling appeal or safety of items in the oral cavity, providing feedback to the digestive system about incoming food, and supporting the regulation of satiety [17]. It has recently been suggested that over-consumption of dietary fat might alter the sensitivity or the expression of taste receptors [10] and that high-fat diet exposure can decrease sensitivity to fatty acid taste in lean participants [18]. These data suggest sensitivity to fatty acid taste may have a significant impact on eating behaviour and long-term dietary intake with important health consequences [19]. Commonly cited causes of obesity include major changes in our food environment [20] which have led to over-consumption of inexpensive, highly palatable, energy-dense, and nutrient-poor foods. Given that fat rich foods have a powerful hedonic appeal, preferences for fatty foods are important contributors to increases in body weight and metabolic disease risk [21].

To date, only a few studies have investigated the relationship between olfactory sensitivity to fatty acids and dietary intake, eating behaviour, or obesity development [13,22]. Human studies have shown that the fat content level of milk can be discriminated by odour alone, however, this ability was shown to have no relationship with BMI or dairy consumption [13]. Stevenson et al., 2016; found that a western-style diet was associated with poor odour identification as well as poor fat discrimination by taste [22]. Other studies have reported that olfaction may be desensitised in individuals who are morbidly obese [23,24]. This may be due to changes in olfactory sensory neurons (OSNs), with a decline of OSNs shown to occur over time during high-fat intake in animal studies [25]. In contrast, another study found participants with obesity had a stronger hedonic response towards the smell of dark chocolate than non-obese participants, rating the odour as significantly more pleasant [26]. Furthermore, there is emerging evidence that variations in human olfactory receptor gene expression can influence eating behaviour, resulting in increased adiposity [27]. Similarly, it has been suggested that individuals who are obese may have a better sense of smell for food odours but not to non-food odours [26,28]. Despite some authors suggesting olfactory cues may be dispensable for the detection of dietary fats [12], it is not clear whether olfactory sensitivity for fatty acid runs in parallel with an individual’s sensitivity to fatty acid taste. 

Recent work has found the ability to detect different levels of fat in a food matrix is related to taste sensitivity by comparing results of a fat ranking task with threshold sensitivity to oleic acid [9]. Similar fat ranking tasks have classified participants as being fat “discriminators” or “non-discriminators”, where non-discriminators consumed greater amounts of dietary fat and had higher abdominal adiposity [29]. Despite mouthfeel perception and the dynamic nature of eating being critical for food acceptance [30], there are still relatively few studies on how mouthfeel perception relates to other sensory attributes such as taste and olfactory modalities [31,32].

In the present study, we designed a series of experiments to investigate the relationships between fatty acid taste, olfaction, and mouthfeel modalities, and how fat taste perception measurements may relate to dietary intake and eating behaviour. The present study aimed to (i) measure oleic acid taste and olfactory detection; (ii) explore links between oleic acid taste, olfaction and mouthfeel perception of fat; and (iii) investigate oleic acid taste detection and associations with eating behaviour, dietary intake, and body composition.

## 2. Materials and Methods 

Fatty acid taste and olfactory detection rate of oleic acid were determined in this study by extending the commonly used three alternative forced choice (3-AFC) procedure testing past the assumed taste threshold level and by carrying out three repeated sessions to increase accuracy and precision. The relationship between these sensory modalities was established as well as comparison with eating behaviour, dietary intake, olfactory detection of *n*-butanol, mouthfeel perception of fat, and body composition.

### 2.1. Participants

Participants included premenopausal, non-pregnant, non-lactating New Zealand (NZ) European women aged 18–45 years living in Auckland, NZ. All participants self-reported being healthy, had no cold or flu symptoms on test days, had no food allergies or intolerances, nor a dislike towards milk, coconut, or dairy based products, were non-smokers and had no medical history or evidence of conditions that could alter gustatory function e.g., undergoing chemotherapy, having diabetes, nor had taken antibiotics over the past three months [33,34]. Participants were recruited using posters, flyers, social media (e.g., Facebook, Twitter) and via email lists (e.g., Massey University staff and student lists). Participants were screened with an online questionnaire to assess the inclusion and exclusion criteria. This study was conducted according to guidelines laid down in the Declaration of Helsinki and all procedures were considered to be low-risk by the Massey University Human Ethics Committee, NZ. Written, informed consent was obtained from all participants prior to participating in the study.

Participants were required to attend three morning sessions in a fasted state at which they were tested on taste, olfactory, and mouthfeel measurements. Participant heights and weights were measured at the first visit using a standardised protocol (Table 1). Body mass index (BMI) was calculated (weight (kg)/height (m^2^)). Body fat percentage was measured at the first visit using bioelectrical impedance (BIA) measurement (InBody230, Biospace Co. Ltd., Seoul, Korea) and standardised techniques [35]. In between study visits, participants were required to complete two online questionnaires to assess dietary intake and eating behaviour.

### 2.2. Stimuli Preparation for Sensory Measurements 

#### 2.2.1. Stimuli for Fatty Acid Taste Measurement

The methodology for taste testing is described in further detail by Haryono et al. [36]. In brief, a milk emulsion vehicle was used and made from non-fat UHT milk (Homebrand, Auckland, New Zealand), added to a glass beaker along with food grade gum arabic (Hawkins Watts, Auckland, New Zealand). To prevent oxidation, 0.01% EDTA (FCC, Spectrum Laboratory Products Inc., Gardena CA, United States) was added. The milk base had 5% mineral oil added (Purity FCC grade, Petro-Canada Canadian Oil Company, Mississauga, ON, Canada). This solution was homogenised thoroughly with a Silverson L4RT homogeniser (Silverson, Longmeadow, MA, United States). The milk base solution was divided in half so that a series of the fatty acid vehicle with increasing concentrations of oleic acid could be created. Half of the milk base was used for the blank testing solutions. Each concentration in the series of active stimuli required a separate beaker. In each beaker in the series, the appropriate amount of oleic acid (Sigma-Aldrich, St. Louis, MO, USA) was added to the milk. Homogenisation of each beaker was undertaken in ascending order. The homogeniser was sanitised after contact with oleic acid to prevent any contamination of non-oleic acid solutions. Testing stimuli were made fresh on the day of evaluation. This oleic acid in milk emulsion concentration series (0.02, 0.06, 1, 1.4, 2, 2.8, 3.8, 5, 6.4, 8, 9.8, 12, and 20 mM) has previously been used in several studies [9,18,37,38]. 

#### 2.2.2. Stimuli for Fatty Acid Olfactory Measurement

**Figure 1:**
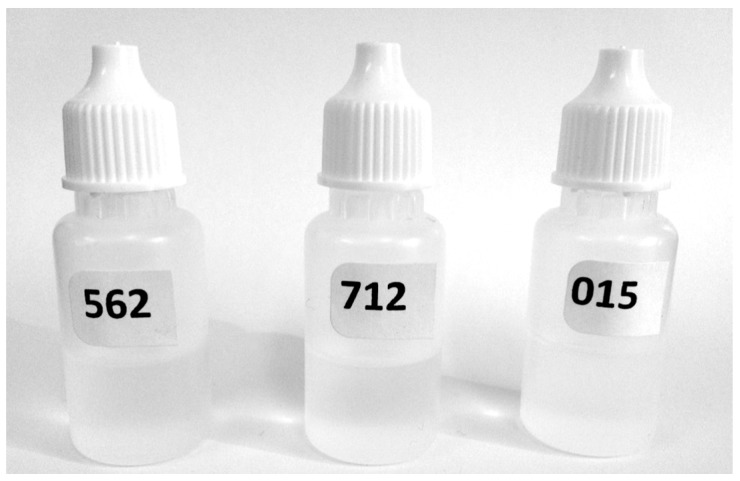
Image of sniffing bottles for oleic acid olfactory measurement using the ascending 3-AFC procedure.

Olfactory stimuli were created from oleic acid to create a series with an increasing concentration of fatty acid content. The stimuli and procedure were developed in order to align with the taste testing [36], however, our internal testing showed that higher concentrations of oleic acid were required for olfactory detection. The stimuli were prepared by adding the oleic acid to odourless light mineral oil (Sigma-Aldrich, St. Louis, MO, USA) in a concentration series. All blank testing solution bottles contained 5 mL of odourless light mineral oil. The series of active stimuli concentrations ranged from a 6 mM oleic acid solution to a 380 mM oleic acid solution (6, 12, 24, 48, 95, 190, and 380 mM). Olfactory stimuli were kept in small, individual containers with a screw top lid (Figure 1). Oleic acid required mixing by drawing three or four times with a 10 mL pipette, to ensure an even emulsion of fatty acid and mineral oil. All olfactory stimuli were made fresh on the day of evaluation. All oleic acid used was from the same batch as the tasting procedure and obtained from the same supplier to allow for comparison across testing stimuli. The methodology for olfactory testing used the same procedure as taste testing (3-AFC) but with a decreased number of concentrations (see Table 1).

#### 2.2.3. *n*-Butanol Threshold Test

The overall olfactory performance of each participant was established using a “Sniffin’ Sticks” test kit which has been widely used in research [39] in order to compare results with the oleic acid olfaction test. The “Sniffin’ Sticks” kit contains 16 pen sets (triplets) with increasing thresholds of the volatile *n*-butanol, alongside blank odour pens (Burghart Instruments, Wedel, Germany). The 16 pen sets require presentation of three pens each time with only one of the three pens containing the target odourant (forced choice procedure). Pen No. 1 is the highest concentration and pen No. 16 is the lowest, with a high score representing increased sensitivity to *n*-butanol, and a score over 6.5 considered to be “normosmia” [39,40].

#### 2.2.4. Subjective Mouthfeel Measurement Test

For testing of mouthfeel and textural influence of fat, vanilla custard tasting stimuli were designed. Test stimuli were created using coconut oil, which has a high saturated fat content providing a high level of fat-related textural attributes [41]. The base for the vanilla custard involved mixing 3 tablespoons of cornstarch (Goodman Fielder Ltd., Auckland, New Zealand), 3 tablespoons of sugar (Homebrand, Auckland, New Zealand), 1 teaspoon vanilla essence (Hansells Food Group, Auckland, New Zealand), ¼ teaspoon of yellow food colouring (Hansells Food Group, Auckland, New Zealand), and 500 mL of non-fat milk (Homebrand, Auckland, New Zealand). The mixture was heated in a microwave (4 min, stirred, 3 min, stirred) and homogenised. Coconut oil (Blue Coconut, Canterbury, New Zealand) was added to each bowl at 0%, 5%, 10%, and 15% quantities and then custard was added to give a total weight of 50 g. Each bowl was thoroughly and consistently mixed. Smaller portion cups (35 mL cups) were labelled with individual three-digit codes and filled with 20 g of custard and then refrigerated. Testing stimuli were made approximately 12 h prior to taste testing (see Table 1 for details of methodology).

Subjective evaluation of vanilla custard was recorded on paper by placing a vertical line through 150 mm linear scales. The questionnaire and overall evaluation used to assess the vanilla custard (real world food model) was similar to that of consumer sensory evaluation techniques [36,51]. At each fat concentration level, a separate questionnaire was administered, with no side-by-side comparisons. Participants were not informed of the fat content levels and were untrained, as we were looking for naive ratings of attributes and ratings of hedonic liking associated with a real world food model [49]. Prior to the assessment, the participant was told that there were no “right or wrong” answers. On each visit, participants evaluated two out of the four custard stimuli. These were selected in a randomised order so that all four custard stimuli were tested at the first two visits. The questions asked related to liking (“How much do you like or dislike the aroma/taste/mouthfeel/sweetness of the vanilla custard?”), ideal preferences (“Compared to your ideal vanilla custard, what do you think of the aroma/flavour strength/mouthfeel/sweetness?”), overall like or dislike (“Overall, how much do you like or dislike this vanilla custard?”), fat content (“How would you rate the fat content level?”), and fat taste intensity (“How would you rate the intensity of the fat taste?)” [21,52]. The scales were anchored at either end by statements; “Strongest imaginable dislike/Too weak/Not enough flavour/Too dry/Not sweet enough/Very low fat content/Very low fat taste” on the left and “Strongest imaginable like/Too strong/Too much flavour/Too fatty oily/Too sweet/Very high fat content/Very high fat taste” on the right. The final question was to circle the % of fat thought to be present in the vanilla custard stimuli from a range of options (0%, 1%, 2%, 5%, 10%, or 15%).

### 2.3. Eating Behaviour and Dietary Intake Questionnaires 

Eating behaviour was assessed using the validated three-factor eating questionnaire (TFEQ) to measure cognitive dietary restraint (21 items), disinhibition of control (16 items) and susceptibility to hunger (14 items) [54]. Each item scores either 0 or 1 point with possible scores ranging from 0-0-0 to 21-16-14 [54]. TFEQ scores were allocated for each category and associated subscales were calculated under each of the three factors [55,56,57], where higher scores denote higher levels of restrained eating, disinhibited eating, and predisposition to hunger [54]. 

Dietary intake (energy, macronutrients, food groups) was measured by a 220-item food frequency questionnaire (FFQ) developed for the Women’s EXPLORE study (“EXamining Predictors Linking Obesity Related Elements”) and adapted from the FFQ used in the National Nutrition Survey in NZ [34,53,58,59]. Approximate frequency of food and beverage intake was for items consumed over the previous month. Dietary data from the FFQ were combined into key food groups as recommended by the Eating and Activity Guidelines for NZ Adults of (i) fruit; (ii) vegetables; (iii) grains; (iv) milk and milk products; (v) nuts, nut spreads, and seeds; (vi) eggs, poultry, and fish; (vii) red meat; (viii) takeaways; (ix) sugary treats; (x) butter, margarine, and oil; (xi) legumes; and (xii) alcohol [60]. Dietary intake data are expressed as Daily Frequency Equivalents (DFEs) where frequency responses were calculated as a per day value (i.e., “4–6 times/week” was calculated as 5/7 days = 0.71 DFE’s [61]. Data from the FFQ was entered into Foodworks version 7, 2012 (Xyris Software Pty Ltd., Kenmore Hills, Queensland, Australia). Foodworks uses the NZ Food Composition Database and FOODfiles [62] to determine total energy (kJ) and macronutrient (g) intake.

### 2.4. Testing Procedure 

Each participant attended three testing sessions which took place on nonconsecutive days within a period of one month. During all three testing sessions, oleic acid taste and olfaction threshold testing was conducted. Participants were asked not to eat, drink, wear perfume, or taste any other products prior to testing and on arrival had fasted for approximately 12 h. Testing took place between 7:00 a.m. and 9:30 a.m. All stimuli were evaluated at room temperature (20 °C) in individual taste testing booths. Tasting stimuli were whole mouth samples which were evaluated using a sip-and-spit procedure with no solution ingested [12]. Participants were asked to wear a nose clip for the taste evaluation only. Prior to sensory testing, a short training procedure was conducted to familiarise each participant with the unique taste of the oleic acid solution (9.8 mM) while wearing a nose-clip and to compare this taste to that of a blank milk emulsion (R.S.J. Keast, [63], June 2015). If the participant failed to notice any difference between the target (9.8 mM) and the blank, they were asked to try again with higher concentrations, until the participant recognised the taste. During the testing procedure, participants rinsed out their mouth with water between each set of tasting stimuli. Oleic acid taste perception testing was conducted first (wearing nose-clip), followed by oleic acid olfactory testing (nose-clip removed). Red lights were left on throughout the tasting, olfactory, and custard stimuli evaluation. To finish, evaluation of vanilla custard tasting stimuli (visit 1 and 2) or the *n*-butanol threshold test (visit 3) were conducted. All testing took approximately one hour to complete. The TFEQ and FFQ were answered in one sitting on SurveyMonkey™ in between study visits in a location where participants had minimal distraction.

### 2.5. Statistical Analysis 

Statistical analyses were performed using IBM SPSS software for Windows (version 22.0; IBM Corp, Armonk, NY, USA). The sample size calculation was performed based on a pilot study which measured linoleic acid fatty acid taste threshold. It was estimated that 50 participants were required based on alpha risk at 0.05 and beta risk at 0.2 (power 80%) to find a difference in oleic acid taste perception between concentration levels. The sample size is similar to other studies investigating oleic acid taste perception [9,31,64]. Binomial regression models were performed using R version 3.2.5 (R Project for Statistical Computing, Vienna, Austria). Data were checked for normality using the Kolmogorov–Smirnov test. Normally distributed data are presented as mean ± SD and non-normally distributed data as median (25th–75th percentiles). Correlations of fatty acid taste detection, *n*-butanol detection, oleic acid olfaction, and dietary intake were tested using Pearson’s correlation coefficient and Spearman’s correlation coefficient depending on the normality of the data. Intra-class correlation (ICC) using two-way random effects model, single measures were performed to detect associations between fatty acid detection thresholds and proportion of correct trials across testing days. The fitted fat taste models of the probability of correct detection used binomial regression to model the success or failure of taste and olfaction detection. Binomial regression employs a link function to connect the binary outcome variable with the continuous predictor variables. The link functions that gave the most stable fit to each data set were applied, these were the logit link function for the olfaction data, and the complementary log–log function for the taste data at all three testing sessions [65]. The rationale to interpolate at 0.66 probability was based on the publication by Lawless, 2010 [65]. Lawless (2010) describes an alternative analysis of forced-choice threshold data sets [65,66]. The interpolation of chance-corrected thresholds does not discount correct responses of early concentrations but does take into account the probability that guessing correctly may have. This analysis does not exhibit the downward bias that can occur from correct guessing and has proven practical applications [65]. 

The probability of guessing would be 0.33 or 1/3 correct trials. Therefore, the probability of 0.66 or 2/3 correct samples ensures that the detection rate is above a “chance” level. By using a single 0.66 probability cut-off for all participants we were able to assess their sensitivity at the same point identified by the binomial regression model. A similar approach has been used by Giguère et al., 2016 and Jayasinghe et al., 2017 [67,68]. The probability of 0.66 differentiated between participants at a common intercept and is a chance-corrected detection rate.

Sensitivity to oleic acid was treated as a grouping variable and was defined as “hypersensitive” or “hyposensitive” to taste similar to previous publications [9,43]. For this purpose, we chose the cut-off of 5.7 mM as this was the median detection threshold on average, across the three days. Group differences between taste detection rate, taste detection threshold, olfactory scores, eating behaviour scores, energy intake, macronutrient intake, food group intake, and mouthfeel ratings (continuous variables) were investigated using independent samples *t*-tests and Mann–Whitney U test for non-normally distributed data. BMI categories and taste sensitivity (hypersensitive versus hyposensitive) were compared using a chi-squared test. A *p*-value of <0.05 was considered as significant.

## 3. Results

### 3.1. Participants

A total of 51 female NZ European participants were recruited to take part in the study (Table 2). Of these, 50 women completed all three required visitations. The majority of the participants were of normal weight (BMI between 18.5 and 24.9 kg/m^2^; 62%), with some overweight (BMI 25–29.9 kg/m^2^; 22%), and some obese (BMI ≥ 30 kg/m^2^; 16%) participants. 

### 3.2. Taste and Olfaction Detection Curves of Oleic Acid

**Figure 2:**
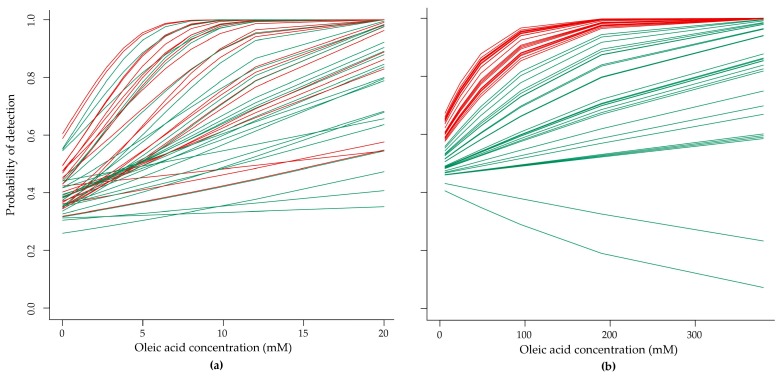
Comparison of (**a**) taste detection curves and (**b**) olfactory detection curves of oleic acid (*n* = 50). Participants marked in red showed strong olfactory detection rate (**b**) and those same participants are shown in the taste model also in red (**a**). The fitted models of binomial regression for taste and olfaction show successful vs. failed individual trials across all three testing days (3-AFC) modelled with a link function.

Detection curves were modelled from the data obtained from all three sessions in order to interpolate the detection rate of oleic acid taste perception for each individual. Each taste trial, which was comprised of a set of three samples containing two controls and one oleic acid “target” sample, was used to create a binomial regression model (incorrect vs. correct identification per 3-AFC set). Figure 2a shows the taste detection curves (Detection curves: concentration of taste stimulus (mM) vs. the probability of correctly identifying taste) of oleic acid using the probability (Probability of detection: the probability of a trial being correctly identified at each concentration (3-AFC method). One in three trials will be correctly identified by chance alone (0.333, or 33.3%). Each line represents an individual participant showing that detection ability increases with increasing concentrations of oleic acid.

We evaluated between session repeatability of the probability of detection (taste) for each participant, where moderate significant correlations were found across all testing sessions 1, 2, and 3 (ICC = 0.52, CI = 0.36–0.67, *p* < 0.001, two-way random effects model, single measures). When comparing side-by-side sessions “1 and 2”, and “2 and 3”, repeatability was stronger, showing significant moderate correlations (ICC = 0.67, CI = 0.48–0.80, *p* < 0.001 and ICC = 0.59, CI = 0.38–0.75, *p* < 0.001, respectively). Detection curves for taste were variable across the group of participants but significantly repeatable within an individual’s data sets across sessions 1, 2, and 3. In addition, there were no significant differences between taste detection thresholds across the three sessions.

Figure 2b shows the olfactory detection curves using the probability of correctly identifying oleic acid odour at each trial. Olfactory detection curves increased with higher concentrations of oleic acid (data obtained from all three sessions). When evaluating the repeatability of the probability of detection for oleic acid odour, moderate correlations were found across visits for sessions 1, 2, and 3 (ICC = 0.41, CI = 0.23–0.58, *p* < 0.001, two-way random effects model, single measures). When comparing side-by-side sessions “1 and 2”, and “2 and 3”, olfactory repeatability was consistent with comparing all three sessions showing significant moderate correlations (ICC = 0.44, CI = 0.19–0.64, *p* < 0.001 and ICC = 0.39, CI = 0.13–0.6, *p* < 0.002, respectively). There were no significant differences between olfactory detection thresholds across the three sessions.

### 3.3. Fatty Acid Taste Hypo- and Hypersensitivity

Previous studies have defined oleic acid taste hypersensitivity empirically as a detection threshold—the lowest concentration at which a stimulus is detected, determined by three consecutively correct taste trials at that given concentration (3-AFC method)—at a concentration of less than 3.8 mM [36]. In this current study, we used a model to determine taste detection rate. Interpolation of each detection curve was required to characterise participants as hypo- or hypersensitive to oleic acid taste perception. In order to create an equivalent classification to previous studies [10,36,37], the concentration (mM) at which detection is 0.66 (66%; equivalent to successfully obtaining two out of three correct trials at that given concentration) was considered to be the detection rate (Detection rate: concentration of stimulus at which an individual would correctly identify two out of three (0.66 or 66%) trials, using the predictive detection rate curves). Taste hypersensitive participants (*n* = 22) were defined as individuals who obtained a detection rate of less than or equal to 5.7 mM at 0.66 (66%) of the trials, based on their taste detection curve (Table 3).

### 3.4. Relationship between Oleic Acid Taste Perception and Olfaction

**Figure 3:**
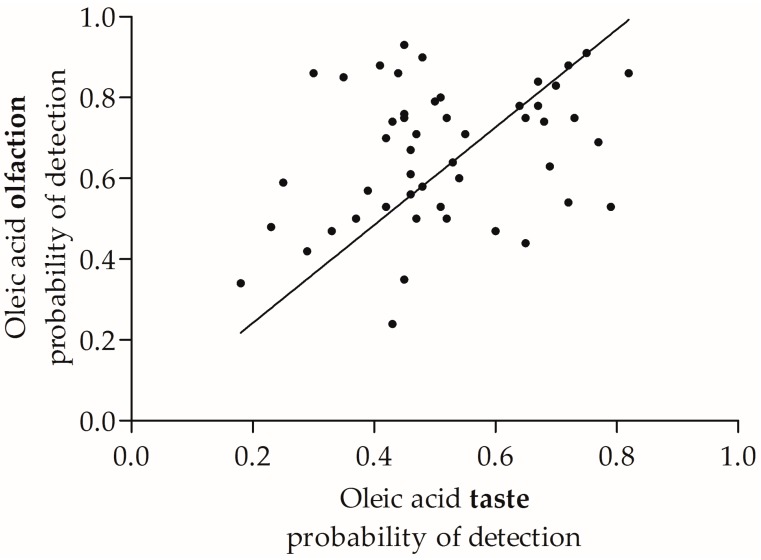
Scatterplot of the relationship between oleic acid olfaction and taste (probability of detection), the weighted average across all three sessions.

A significant, positive correlation between taste probability of detection and the olfactory probability of detection (*r* = 0.325, *n* = 50, *p* < 0.02) of oleic acid was found (Figure 3).

### 3.5. Relationship between Oleic Acid Taste Hypo- and Hypersensitivity, Olfaction Detection Rate, n-Butanol Olfactory Threshold, and Eating Behaviour 

Taste hypersensitive participants had a lower olfactory detection rate (higher sensitivity) than the hyposensitive taster group (Table 4, *p* < 0.05). Scores obtained for *n*-butanol threshold demonstrated a similar relationship to oleic acid olfactory detection rate, taste hypersensitive participants obtained a higher mean score (higher sensitivity) for *n*-butanol threshold “Sniffin’ Sticks” (*p* < 0.03). The mean score for *n*-butanol threshold was 8.7 ± 2.2. Based on normative values, 43 participants were classified as normosmic (test score > 6.5), and 7 as hyposmic to odour (test score < 6.5, less sensitive). There was a trend for the olfactory oleic acid detection rate (mM) to correlate with *n*-butanol threshold score (*r*_s_ = −0.263, *p* = 0.07). The three eating behaviours assessed by the TFEQ were cognitive restraint, disinhibition, and hunger as well as associated subscales. Results from the questionnaire were analysed based on scoring criteria established by Stunkard and Messick (1985) [54]. For cognitive restraint, the majority of participants (68%) reported low scores (0–10 out of a possible score of 21). Participants also reported mostly low scores for disinhibition (74% scored 0–8 out of 16). For susceptibility to hunger, 80% of participants scored low (0–7 out of 14) [69]. A significant difference in disinhibition score and emotional disinhibition (subscale) was observed between hypersensitive and hyposensitive groups (*p* < 0.05; *p* < 0.03, respectively). There were no significant correlations between eating behaviour factors when compared to oleic acid olfactory detection or *n*-butanol threshold (all, *p* > 0.05).

### 3.6. Relationship between Oleic Acid Taste Hypo- and Hypersensitivity, Mouthfeel Rating, and Olfaction

**Figure 4:**
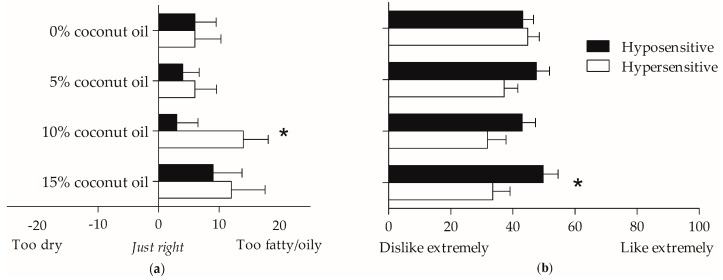
Comparison of (**a**) mouthfeel rating and (**b**) mouthfeel liking of high (15% coconut oil), medium (10% coconut oil), low (5% coconut oil) and no fat custard (0% coconut oil) split by taste hypersensitive (*n* = 22) and hyposensitive (*n* = 28) participants. Data presented as mean ± sem. * *p* < 0.05.

Distinct from fatty acid taste is the ability to feel the texture of fat in food or drinks in the mouth, which are the tactile sensations that can be described as “creamy” or “oily” [41]. Significant differences were found between hyposensitive and hypersensitive participants when asked to subjectively rate how much they liked the mouthfeel of high-fat custard (*p* < 0.05) and when rating the mouthfeel of the medium fat custard in comparison to what they perceived as an ideal level of fat content (*p* < 0.05) (see Figure 4). Additional vanilla custard rating questions (e.g., sweetness intensity, flavour liking, etc.) were not significantly different between hyper- and hyposensitive taste groups (all, *p* > 0.05).

Mouthfeel perception of high-fat custard (15% coconut oil) was correlated with *n*-butanol sensitivity, where a rating of “too fatty/oily” was associated with higher olfactory sensitivity (*r* = 0.393, *p* < 0.01). No other noteworthy significant associations were found between the mouthfeel ratings of custard, *n*-butanol threshold or oleic acid olfactory threshold. 

### 3.7. Relationships between Oleic Acid Taste Perception, Oleic Acid Olfaction, Dietary Intake, Mouthfeel Rating, and Eating Behaviour 

Nuts, nut spreads, and seeds food group intake (DFE’s) was significantly correlated with oleic acid olfactory detection rate (mM), where high sensitivity (low detection rate) correlated with higher intake of nuts, nut spreads, and seeds (*r*_s_ = −0.410, *p* < 0.01). We found no other significant relationships between oleic acid olfactory detection rate, *n*-butanol threshold, or oleic acid taste perception and food group intakes, energy and macronutrient intake. Additionally, there were no significant differences in food groups, energy, or macronutrient intake between hypo- and hypersensitive taste groups. 

Restraint and hunger eating behaviour factors were related to intake of specific food groups in our sample population. A high hunger score was correlated with higher intake of takeaways (*r*_s_ = 0.33, *p* < 0.02) and butter, margarine, and oil (*r*_s_ = 0.32, *p* < 0.03). High-sugar treat food intake was inversely associated with restraint (*r*_s_ = −0.39, *p* < 0.01). Vegetable intake was positively correlated with restraint score (*r*_s_ = 0.32, *p* < 0.03) and negatively with hunger score (*r*_s_ = −0.31, *p* < 0.03). No other significant relationships between eating behaviour, food group intake, energy, or macronutrient intakes were found.

### 3.8. Oleic Acid Taste Perception and Olfaction Detection Rate and Body Composition

Oleic acid taste hypersensitive participants were significantly more likely to have a low BMI (be lean) (*X*^2^ (1, *n* = 50) = 3.89, *p* < 0.05) and hyposensitive participants were 3.4-times more likely to be overweight or obese (BMI ≥ 25 kg/m^2^) than hypersensitive participants. There was a trend for hypersensitive participants to have a lower PBF than hyposensitive participants (27.8% ± 7.2 vs. 32.2% ± 8.8; *p* = 0.06). There were no relationships found between oleic acid olfactory detection rate and BMI as a continuous variable or between BMI categories or percentage body fat. There were no significant differences in oleic acid olfactory detection rate, oleic acid mouthfeel perception, *n*-butanol threshold, food group intake, energy, macronutrient intake, or eating behaviour between BMI categories.

## 4. Discussion

The present study investigated the relationship between oleic acid taste and olfaction detection rates, and how these measurements may relate to dietary intake, eating behaviour, mouthfeel ratings of fat added to test custard, and body composition. The results show that sensitivity to oleic acid taste perception and olfaction varies considerably between participants, with individual detection rates covering three orders of magnitude. The present study shows for the first time that fatty acid olfactory sensitivity is clearly linked with fatty acid taste sensitivity albeit acting through separate pathways. Hyposensitivity to fatty acid taste was associated with disinhibited eating behaviour. Furthermore, participants who were hypersensitive to oleic acid taste perception had lower BMI values than those who were hyposensitive. The findings of this study show remarkable parallels in fatty acid taste and olfaction detection rates with clear and consistent individual differences in detection ability. These individual differences in fat detection appear to be linked with disinhibited eating behaviour that may have implications for long-term metabolic health outcomes [70]. 

### 4.1. Oleic Acid Taste and Olfactory Detection Rate

In this study, we created tailored models to characterise the detection curves of taste and olfaction with increasing oleic acid concentrations in healthy women. Our data confirm that there is great variability in taste sensitivity between individuals, which is consistent with previous studies in presenting a range of taste detection thresholds across different participants [8,9,31]. In humans, sensing of “fat” has been attributed to CD36 receptors in taste cells, as well as GPR120, 41, 40, and 43 receptors [71,72,73]. It is thought that CD36 receptors may function in fatty acid recognition at low concentrations, whereas GPR120 may be functioning at higher concentrations, acting to enhance the signalling of fatty acids and providing sustained taste experiences [74]. Thus, oral detection of fatty acids may be a result of dual, complementary mechanisms [75]. We therefore chose to identify the probability of oleic acid taste detection over a wider range of concentrations in the present study, given that fat taste may be detected by multiple receptors and a range of transduction pathways [17,74,75,76,77]. 

In consideration of a postulated multiple receptor mechanism that detects fatty acids, we chose to extend the ascending 3-AFC method to continue testing past three correct evaluations, by adding an additional three higher concentrations past the commonly used “stopping point” [46]. An extension of the stopping rule was further implemented to collect enough data points across the three repeated sessions to create the binomial regression models. We were then able to interpolate an individual’s performance using the model and from this, we were able to classify individuals as hypo- or hypersensitive as a grouping variable. This approach decreases the number of false-positives which can occur through guessing the correct solution by chance alone [45]. The extension of the stopping rule further enhanced the quality of our data by broadening the range of concentration levels evaluated, which allowed us to model taste behaviour for each participant. One of the limitations of extending the procedure is inducement of fatigue, but the integration of the probability of correct detection at each concentration level obtained from multiple visits decreases the influence of this effect on the detection rate [45,46]. The fatty acid detection rates applied in this study account for the possibility of guessing correctly but do not discount the correct responses which may occur at low concentrations. The between-participant variance was further reduced in this study by limiting our participants to the same gender, age range (premenopausal only), and to one ethnic group. 

### 4.2. Fatty Acid Taste Hypo- and Hypersensitivity

We were able to identify a detection rate for all participants to then further establish our classification into hypo- or hypersensitive fatty acid taster groups, based on their performance across three days of testing, as opposed to a single session measurement. The ratio of participants classified as hypersensitive in our study, based on their detection rate, was comparable to findings in previous studies [10,37]. The repeatability of fatty acid taste threshold assessments has been investigated previously [44,78], and consistent with these studies, we found significant repeatability across all sessions. Whilst we found that fatty acid taste detection was clearly repeatable, we would recommend a minimum of three testing sessions to measure fatty acid taste or olfaction detection rates, in order to obtain enough data to determine the probability of correctly identifying taste at each concentration level [44,64,78]. In the present study, all sessions were conducted in the morning, prior to consuming breakfast, which we believe enhanced the repeatability of the taste perception data in this study.

### 4.3. Relationship between Oleic Acid Taste Perception and Olfaction

Our results show that the ability to detect fatty acid by taste was significantly associated with that of olfaction. In a “real-world” food setting, the recognition of fat taste would be further enhanced by mastication behaviour (chewing), due to the enhanced release of organic odour volatiles [79]. In support of a fatty acid taste and olfactory relationship, the previous research identified that the expression of the CD36 receptors in the olfactory epithelium may be related to the long-chain fatty acid taste receptor mechanisms [80]. There is a possible role in odorant detection by this scavenger receptor [80], suggesting individuals with higher CD36 taste expression potentially have a homologous olfactory detection ability. This has been observed in CD36-deficient mice who displayed altered olfactory behaviour when exposed to long-chain fatty acids [80]. In support of our findings, in vitro work on human olfactory mucus has found an odorant binding protein which has a strong affinity for long-chain fatty acids, including lauric acid and capric acids [80]. Additionally, the variability in a human odorant-binding protein OBPIIa was associated with individual differences in the bitterness perception of oleic acid [77]. It would be interesting to investigate in future studies whether individuals who are more sensitive to oleic acid olfaction are carriers of the variation in the olfactory binding protein described by Tomassini Barbarossa et al. [81]. Interestingly, in humans, olfactory-based discrimination of the fat content of milk and specific fatty acids at a range of concentration levels has been demonstrated and supports the notion that humans are able to detect small differences in fat content by odour alone [13,47]. 

In comparison to the oleic acid tasting procedure in this study, the concentration of oleic acid used in the olfactory detection tests went to a considerably higher concentration. This was required due to the stimuli being tested orthonasally, at room temperature (20 °C), with fresh oleic acid in partially filled bottles to generate an open headspace for inhalation. Because this is a new procedure we covered a wider range of concentrations but used fewer steps (seven concentrations) as we wanted to avoid adaptation effects which have been reported in some previous olfactory studies [82,83]. 

Our results indicate that there is a difference between hypo- and hypersensitive groups in sensitivity to *n*-butanol odour (“Sniffin’ Sticks” score). The *n*-butanol odour sensitivity test is widely used for the evaluation of human olfactory performance and can be used by medical practitioners to assess olfactory dysfunction [40]. In the present study, the *n*-butanol threshold test was incorporated to see if a well-established olfactory assessment method may relate to the oleic acid olfactory test introduced in this study. We found that *n*-butanol sensitivity is weakly associated with oleic acid olfactory perception. 

### 4.4. Oleic Acid Taste Perception and Disinhibited Eating Behaviour

Significant associations were found between oleic acid taste sensitivity and “disinhibition” and the eating behaviour sub-category “emotional disinhibition”, where higher disinhibition scores were obtained by the oleic acid taste hyposensitive group. Disinhibition refers to opportunistic eating behaviour, which could play a role in weight gain [84]. Previous studies have found emotional disinhibition significantly predicts body fat percentage in young NZ women [85], and another study in a young French cohort found higher disinhibition scores were associated with a higher BMI [86]. It has been suggested that an inability to detect fat efficiently may result in compensation of cognitive satisfaction with other tastes, such as “sweet” [87] which may account for additional weight gain over time [88,89]. Eating behaviour and fatty acid taste sensitivity have not been directly compared in previous studies [29,38,69]. This study is the first to report a relationship between fatty acid taste sensitivity and disinhibited eating behaviour. 

### 4.5. Oleic Acid Taste Perception and Mouthfeel

The subjectively rated mouthfeel of fat in the food matrix in the present study varied between hypo- and hypersensitive participants. Given the textural properties of fat, it was important to investigate whether fatty acid taste sensitivity (which is independent of texture or mouthfeel) can be compared to the liking of “real-world” foods. In this study, we found that oleic acid taste hypersensitivity was significantly related to increased rating of “oily/fatty” mouthfeel perception, as well as negatively impacting the hedonic liking of a high-fat product. The hedonic liking of fat textural attributes in comparison to fatty acid taste sensitivity was of interest, as it is well known that commercial products with “fat replacers” often fail to attain the craving response of a full fat equivalent [90]. This study provides further evidence that fat taste itself (i.e., the presence of fatty acid ligands), in addition to mouthfeel, plays a critical role in the recognition and perception of fat. It is recognised that the presence of fatty acid ligands are critical throughout the digestion process as receptors in the gut are considered to be homologous with oral taste receptors, which may further support an individual’s satiety response [91]. In animal studies, it has been shown that consumption of a high-fat diet related to an increase in CD36 mRNA expression [92]. CD36 mRNA expression was found to occur on circumvallate papillae as well as duodenal enterocytes, supporting the possibility that there is complementary sensing of long-chain fatty acids in the two different regions [92]. Receptors isolated from human intestinal enteroendocrine cells include CD36 and G-protein coupled receptors (GPR120, GPR40) [93]. The CD36 protein, in particular, is expressed in the duodenum and jejunum and has been proposed to play a role in signaling pathways that mediate fatty acid detection in the gut [91,93]. 

### 4.6. Oleic Acid Taste Perception, Olfaction, and Body Composition

The results of the present study showed that participants who were hypersensitive to oleic acid taste had a significantly lower BMI, a finding consistent with previous studies on oleic acid taste perception [9,10,87,94] and linoleic acid taste perception [21]. Positive correlations have also been found between fat preference scores and percent body fat estimates [95]. A comprehensive review by Cox et al. concluded that low sensitivity to fat taste, as well as liking and preference for fat, is related to higher weight status [19]. However, not all studies have found an association between taste sensitivity and BMI [31,96,97]. A recent meta-analysis of studies on taste sensitivity has concluded that fatty acid taste sensitivity does not precede or result in obesity [98]. Of particular interest are studies which have found that a low-fat diet or a high-fat diet can modulate taste sensitivity, where a low-fat diet was shown to significantly increase taste sensitivity to oleic acid over a four-week period while there was no significant difference in sensitivity at baseline [18]. In support of this, a six-week, low-fat dietary intervention study in overweight and obese participants showed that fat taste sensitivity can be enhanced significantly during this time period [99]. These studies support the notion that fat taste sensitivity can be related to body fat mass in some settings. 

Our results did not suggest any direct relationship between oleic acid olfactory sensitivity and body composition. Interestingly, a report by Fernandez-Garcia et al. [100] suggests that olfactory function may be desensitised in response to changing levels of endocrine regulation in the obese state [101]. Increased visceral body fat functions as an endocrine gland with increased secretion of adipokines [100]. Another recent study has found that a decreased sense of both taste and olfaction correlated with visceral fat rating [100]. However, in patients that have had gastric bypass surgery, olfactory function does not change, while in contrast taste sensitivity can improve [102]. Our data further suggests that overall olfactory sensitivity is not directly linked to eating behaviour or dietary intake. In support of this, a previous study found that milk odour discrimination performance was not related to BMI [13]. Future studies focusing on sensory sensitivities across different BMI categories are required to explore the relationship between taste and odour sensitivity in conjunction with metabolic health status. It is important to note that body fat percentage values are clinically more relevant than BMI categories [103]. In the present study, we obtained body fat percentage values from BIA measurements, however, it has been shown that typically a BIA will underestimate body fat percentage by 2% [35]. We would recommend that future studies ascertain body fat percentage, ideally from air displacement plethysmography (ADP) or dual-energy X-ray absorptiometry (DXA), and compare these values to chemosensory perception.

### 4.7. Oleic Acid Taste Perception, Olfaction, and Dietary Intake

In this study, no major associations were found between taste sensitivity and dietary intake. However, we did find a significant association between sensitivity to olfactory oleic acid and the intake of “nuts, nut spreads, and seeds”. Olfaction has been identified as an important means for the interpretation of food flavours, and hedonic liking is due to the presence of odour volatiles released during the eating process [79]. It is possible that participants who had a higher intake of nuts and seeds may have an increased ability to recognise the associated odours. To date, there has been no consensus about whether there is a relationship between fat taste sensitivity and dietary intake. It is likely that discrepancies between studies are due to differences in the study participants (e.g., gender, ethnicity, age), assessment methods of fat taste perception (psychophysical measurement, type of fatty acid stimulus) or dietary intake assessment methods which in turn generates inconsistencies about the potential biological or functional relationships [19,31]. The FFQ used in this study is a retrospective account of dietary intake, which was used to obtain individual energy (kJ), macronutrient (fat, protein, carbohydrate, and saturated fat), and food group intakes. A recommendation for future studies would be to measure dietary intake with a four-day food diary directly prior to taste testing. Whilst all self-reported dietary intake assessments are influenced by under- or over-reporting [104], we consider the FFQ would be better used as a population tool for larger studies as opposed to individual comparisons to physiological mechanisms.

### 4.8. Additional Strengths and Limitations of this Study

In this study, the sample size was powered for determining significant differences in oleic acid taste perception. The additional aspects investigated in the study (e.g., mouthfeel ratings, eating behaviour, BMI, etc.) were exploratory variables that were ancillary to the modelling of fatty acid taste perception. In order to extend any of the findings from this study to the wider population, an incorporation of additional participants and representative demographic groupings would be required. 

## 5. Conclusions

Fatty acid taste detection mechanisms are complex and cannot be explained by a single receptor mechanism [73]. Therefore, the methodology chosen for this study optimised taste perception measurements by detection rate of a single fatty acid across a broad range of concentrations, which modelled each participant’s individual taste behaviour. The modelling of taste behaviour was based on the probability of correctly identifying the oleic acid taste at each concentration level, which was unique to this study and was a refined version of previously applied approaches. Furthermore, we were able to apply the same binomial regression model to olfactory detection which allowed us to compare the chemosensitivity of each sensory modality. This study is the first to report a link between fatty acid taste and olfaction sensitivity in humans. Furthermore, we drew conclusions about specific characteristics of disinhibited eating behaviour in hypo- and hypersensitive fatty acid taste groups which were determined from taste detection rate. 

Although the ability to perceive fatty acid taste varied markedly between participants, the association between fat taste perception and disinhibited eating behaviour observed in the present study suggests that fat taste perception may influence dietary habits that have long-term metabolic health consequences. Additionally, sensitivity to fatty acid taste was related to body composition and hyposensitivity to oleic acid was clearly associated with a higher BMI. In conclusion, our study presents strong evidence for a link between oleic acid taste perception, olfactory perception, and the mouthfeel perception of fat; suggesting there are intimate relationships between multiple modalities of fat sensation in humans. Further research is required to investigate whether there is a causal relationship between fat taste perception and olfaction in the etiology of obesity, especially in an obesogenic environment of highly palatable energy-dense and nutrient-poor foods.

## Figures and Tables

**Table 1:** Trial measurements and methods.

Measurement	Methods	Reference	Equipment	Outcomes
Body composition profile	Anthropometric measurements (height, weight) and BIA	Ling et al., 2011; von Hurst et al., 2015 [35,42]	Direct segmental measurement (DSM) BIA (InBody230, Biospace Co. Ltd., Seoul, Korea). Stadiometer	Body composition -BMI profiling (height and weight) -fat and lean body mass
Taste perception oleic acid (C18:1)	3-AFC procedure ascending method with six correct responses (three at the same concentration and three at consecutively higher concentrations)	Developed in this study with reference to Haryono et al., 2014; Mattes, 2007; Keast et al., 2014; Running, 2014; Stewart et al., 2010; Stewart, Feinle-Bisset, and Keast, 2011; Stewart, Newman, et al., 2011; Tucker and Mattes, 2013 [9,10,36,37,43,44,45,46]	Silverson homogeniser (L4RT)	Sensitivity to oleic acid (C18:1) threshold measurement. Identification of “hypo” or “hypersensitivity”
Olfactory perception oleic acid (C18:1)	3-AFC procedure. Maximum of seven concentration levels	Developed in this study with reference to Boesveldt and Lundström, 2014; Hummel, Sekinger, Wolf, Pauli, and Kobal, 1997; Kallas and Halpern, 2011 [13,47,48]	-	Sensitivity to oleic acid (C18:1) olfactory threshold measurement
*n*-butanol olfactory perception	3-AFC procedure. 16 concentration levels presented in rising order (pens 16, 14, 12, etc.)	Denzer et al., 2014; Hummel et al., 2007, 1997 [39,40]	Burghart Sniffin’ Sticks smell test	Sensitivity to *n*-butanol (Sniffin’ Sticks) olfactory threshold
Mouthfeel perception	Subjective hedonic and intensity linear scales, JAR scales	Developed in this study with reference to Ares, Barreiro, and Giménez, 2009; Keller et al., 2012; Martínez-Ruiz et al., 2014; Popper, 2014; Worch, Lê, Punter, and Pagès, 2012 [21,49,50,51,52]	-	Subjective rating of mouthfeel (intensity, liking, etc.)
Dietary intake	220-item FFQ	Kruger et al., 2015; Houston, 2014 [34,53]	Analysis using Foodworks 7 2012 (Xyris Software Pty Ltd., Kenmore Hills, Queensland, Australia). Questionnaire completed on SurveyMonkey™ online platform	Daily energy, macronutrient and food group intake
Eating behaviour	TFEQ	Stunkard and Messick, 1985 [54]	Questionnaire completed on SurveyMonkey™ online platform	Restraint, disinhibition, and hunger measurement

Abbreviations: BIA, bioelectrical impedance; BMI, body mass index; AFC, alternative-forced choice; FFQ, food frequency questionnaire; JAR, just about right; TFEQ, three-factor eating questionnaire.

**Table 2:** Anthropometric characteristics of participants.

Variable	All (*n* = 50)
Age (year) ^1^	26 (22, 32)
Height (cm) ^2^	166 ± 6
Weight (kg) ^1^	67 (57, 76)
BMI (kg/m^2^) ^1^	24 (21, 28)
PBF (%) ^2^	30 ± 8

Abbreviations: y, years; PBF, percentage body fat; SD, standard deviation. ^1^ Median (25th–75th percentiles); ^2^ Values are means ± SD.

**Table 3:** Comparison of median (25th–75th percentiles) detection rate and detection threshold of hypersensitive and hyposensitive taste groups.

Variable	Hypersensitive (*n* = 22)	Hyposensitive (*n* = 28)	*p*-Value
Detection rate ^1^	3.36 mM (2.14, 5.53)	12.12 mM (8.91, 19.37)	<0.001
Detection threshold ^2^ [10,36,37]	2.58 mM (1.47, 3.35)	11.10 mM (6.07, 12.73)	<0.001

^1^ Detection rate: concentration of stimulus at which an individual would correctly identify two out of three (0.66% or 66%) of trials, using the predictive detection rate curves; ^2^ Detection threshold: the lowest concentration at which a stimulus is detected, determined by three consecutively correct taste trials at that given concentration (3-AFC method).

**Table 4:** Comparison of TFEQ scores and olfactory detection for hyper- and hyposensitive taste groups.

	Hypersensitive (*n* = 22)	Hyposensitive (*n* = 28)	TOTAL (*n* = 50)	*p*-Value
Oleic acid olfactory detection rate ^1^ (mM) ^2^	24.2 (11, 61)	97.3 (24, 181)	45.4 (16, 158)	0.041 ^4^
*n*-butanol threshold score ^3^	9.5 ± 1.8	8.1 ± 2.3	8.7 ± 2.2	0.029 ^4^
Cognitive dietary restraint ^2^	8.0 (4, 11)	10 (7, 12)	9.0 (5, 11)	0.232
Flexible restraint ^2^	3.0 (1, 4)	3.5 (2, 5)	3.0 (1.8, 4)	0.159
Rigid restraint ^2^	2.0 (1, 3)	3.0 (1.5, 4)	3.0 (1, 4)	0.133
Disinhibition ^2^	4.0 (3, 6)	6.5 (3, 10)	5.0 (3, 9)	0.046 ^4^
Habitual susceptibility ^2^	0.0 (0, 1)	0.5 (0, 2)	0.0 (0, 1)	0.197
Emotional susceptibility ^2^	0.0 (0, 1)	2.0 (0, 3)	1.0 (0, 2)	0.029 ^4^
Situational susceptibility ^2^	2.0 (2, 4)	3.0 (1, 4)	3.0 (1, 4)	0.538
Hunger ^2^	3.5 (2, 6)	4.0 (2, 7.5)	4.0 (2, 6.3)	0.313
Internal locus ^2^	2.0 (0, 3)	2.0 (1, 3)	2.0 (0, 3)	0.638
External locus ^2^	1.0 (0, 2)	2.0 (1, 4)	1.5 (0.8, 3)	0.125

Abbreviations: TFEQ, three-factor eating questionnaire; mM, millimolar; SD, standard deviation. ^1^ Detection rate (mM), defined as the concentration at which correct detection is 0.66 (66% correct trials over three days) using the odour detection curves; ^2^ Median (25th–75th percentiles); ^3^ Values are means ± SD; ^4^ Significant difference found between hypersensitive and hyposensitive taste groups (*p* < 0.05).
